# Nicotinamide *N*-Methyltransferase as Promising Tool for Management of Gastrointestinal Neoplasms

**DOI:** 10.3390/biom12091173

**Published:** 2022-08-24

**Authors:** Valentina Pozzi, Roberto Campagna, Davide Sartini, Monica Emanuelli

**Affiliations:** 1 Department of Clinical Sciences, Polytechnic University of Marche, 60020 Ancona, Italy; 2 New York-Marche Structural Biology Center (NY-MaSBiC), Polytechnic University of Marche, 60131 Ancona, Italy

**Keywords:** nicotinamide *N*-methyltransferase, esophageal cancer, gastric cancer, hepatic cancer, pancreatic cancer, colorectal cancer, molecular biomarker

## Abstract

Gastrointestinal (GI) neoplasms include esophageal, gastric, colorectal, hepatic, and pancreatic cancers. They are characterized by asymptomatic behavior, being responsible for diagnostic delay. Substantial refractoriness to chemo- and radiotherapy, exhibited by late-stage tumors, contribute to determine poor patient outcome. Therefore, it is of outmost importance to identify new molecular targets for the development of effective therapeutic strategies. In this study, we focused on the enzyme nicotinamide *N*-methyltransferase (NNMT), which catalyzes the *N*-methylation reaction of nicotinamide and whose overexpression has been reported in numerous neoplasms, including GI cancers. The aim of this review was to report data illustrating NNMT involvement in these tumors, highlighting its contribution to tumor cell phenotype. Cited works clearly demonstrate the interesting potential use of enzyme level determination for both diagnostic and prognostic purposes. NNMT was also found to positively affect cell viability, proliferation, migration, and invasiveness, contributing to sustain in vitro and in vivo tumor growth and metastatic spread. Moreover, enzyme upregulation featuring tumor cells was significantly associated with enhancement of resistance to treatment with chemotherapeutic drugs. Taken together, these results strongly suggest the possibility to target NNMT for setup of molecular-based strategies to effectively treat GI cancers.

## 1. Gastrointestinal Cancers

**Figure 1:**
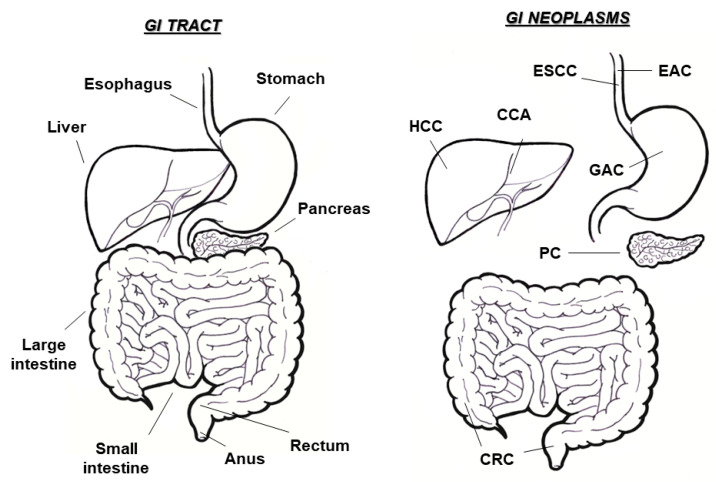
Gastrointestinal (GI) tract and related neoplasms. Schematic representation of the digestive system and overview of main GI cancers, occurring in different anatomic sites (ESCC, esophageal squamous cell carcinoma; EAC, esophageal adenocarcinoma; GAC, gastric adenocarcinoma; CRC, colorectal cancer; HCC hepatocellular carcinoma; CCA, cholangiocarcinoma; PC, pancreatic cancer).

Gastrointestinal (GI) tumors may occur in all anatomic sites within the digestive tract, including esophagus, stomach, liver, pancreas, small intestine, colon, and rectum (Figure 1). They comprise the most frequent malignancies worldwide and contribute around half of the total tumor-related deaths [1]. In particular, the number of detected GI neoplasms constitute more than 20% of the total cancer diagnoses (4 million, of the 14 million subjects annually diagnosed with cancer, are affected with GI tumors), with the incidence of GI tumors being even higher than that combined between breast and lung cancer [2,3].

Esophageal cancer (EC) ranks eighth among all tumors and represents the sixth most frequent cause of cancer-associated death in the world [2]. Because of its asymptomatic behavior, EC undergoes late diagnosis, thus leading to poor patient outcome. For this reason, EC is a very aggressive neoplastic form among GI cancers, displaying an elevated mortality-to-incidence ratio, with a predicted 455,800 new EC diagnoses and 400,200 EC-related deaths worldwide in 2012 [4]. The main risk factors for EC are obesity, smoking habits, alcohol consumption, gastroesophageal reflux disease, and Barrett’s esophagus [5]. The most common EC subtypes are squamous cell carcinoma (ESCC), arising from cells of the inner lining of the esophagus and responsible for 90% of cases, and adenocarcinoma (EAC), that originates from gland cells producing mucous and accounts for 10% of diagnoses [6,7].

Gastric cancer (GC) is the sixth most prevalent neoplasm worldwide. In detail, more than 1,000,000 GC diagnoses occurred and 783,000 deaths due to this malignancy in 2018 [1,2]. Similarly to what was reported above for EC, GC is frequently diagnosed at advanced stage. Therefore, following surgical resection and chemotherapy, associated or not with radiotherapy, most of GC patients experience disease relapse, undergoing poor prognosis [8,9]. Major GC risk factors are sustained Helicobacter pylori infection, male gender, smoking, family history, and use of food preservatives. Among different GC histological subtypes, adenocarcinoma (GAC) represents the most prevalent one, recurring in approximately 95% of stomach malignancies [10].

Although representing a very large organ, as well as the longest part of the GI tract, small bowel (SB) rarely undergoes malignant transformation, with SB tumors accounting for approximately 5% of all GI cancers [11]. On the contrary, GI neoplasms originating from the large bowel (LB) are significantly more frequent and are defined colorectal cancers (CRCs), since they collectively include colon, rectal, and anal tumors. Among human malignancies, CRC is the third most frequent one and represents the fourth most common cause of cancer-associated mortality, being responsible for almost 2,000,000 cases and 900,000 deaths, worldwide [1,2]. Although preventive screening campaigns are active in many countries, half of the patients with early-stage disease proceed to regional or distant metastases and one fourth of CRC-diagnosed subjects even suffer from an advanced tumor form [12,13]. In particular, one third of CRC patients develop hepatic metastases. Due to failure of most anticancer therapies, the prognosis of patients with metastatic CRC is poor and approximately 90% of subjects with late-stage disease die within 5 years [13,14]. In addition to known risk factors, such as cigarette smoking, diet, sedentary life, and obesity [15], CRC development may be promoted by the dynamic imbalance between host immune system and intestinal microbiota [16,17]. Colorectal adenocarcinoma (CRAC) constitutes the most frequent CRC subtype, representing 98% of all cases [18].

The most frequent liver-associated cancers are hepatocellular carcinoma (HCC) and cholangiocarcinoma (CCA). HCC is a primary liver tumor, originating from hepatocytes and accounting for 7% of all cancers worldwide. It represents the most commonly diagnosed liver cancer and displays an aggressive nature, thus leading to a significantly high recurrence rate, as well as to a poor patient prognosis [1,19]. The main HCC risk factors are alcohol consumption and the accumulation of toxic substances and infections sustained by hepatitis B and hepatitis C virus, with 75% of HCC cases being attributable to viral hepatitis [20]. CCA is the second most recurrent neoplasm in liver but is considered a rare cancer form in the world. Due to high metastatic potential and resistance to chemotherapy, it is associated with extremely poor outcomes [21]. Although patients affected with early-stage CCA can undergo transplantation and surgical resection following chemotherapeutic drug treatment with curative intention, mean 5-year survival rate of subjects treated with surgery is around 30% [22,23,24].

Pancreatic cancer (PC) is a very aggressive tumor, associated with a 5-year survival rate of patients below 10%. Due to its increasing incidence, it is estimated that PC will rank in second place regarding mortality causes due to tumors in the next few years [3,25]. The combination of delay in diagnosis and the aggressive behavior makes these neoplasms difficult to be successfully treated. Indeed, metastatic disease often recurs at first detection and even small-sized tumors display high invasive potential, leading to inefficacy of most of treatment strategies [26,27,28].

Due to a delay in diagnosis, most GI cancers are detected at advanced stages and metastatic disease represents the major cause of death for patients affected with these tumors [29]. Therefore, the identification of novel targets for setup of effective therapeutic strategies is urgently required.

## 2. Nicotinamide *N*-Methyltransferase

**Figure 2:**
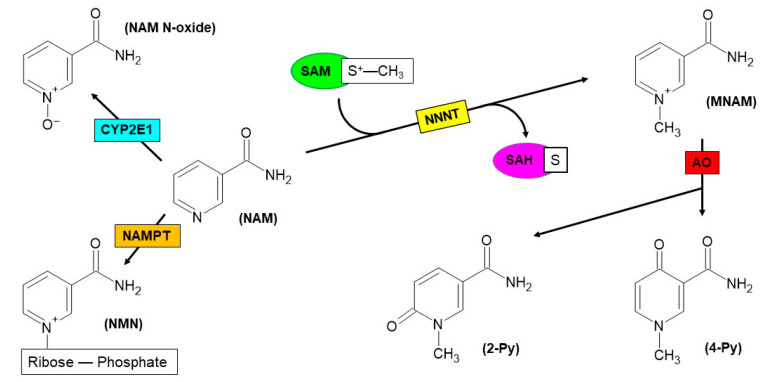
Reactions featuring different fates of nicotinamide (NAM). NAM is first subjected to methylation of nitrogen within the pyridine ring, via the catalysis of nicotinamide *N*-methyltransferase (NNMT) enzyme. S-adenosyl-L-methionine (SAM) is used as a methyl donor and further is converted to S-adenosyl-L-homocysteine (SAH) upon catalysis. Subsequently, N1-methylnicotinamide (MNAM) can act as a substrate of aldehyde oxidase (AO), leading to the formation of oxidation products, such as N1-methyl-2-pyridone-5-carboxamide (2-Py) or N1-methyl-4-pyridone-3-carboxamide (4-Py). An alternative catabolic pathway for NAM is represented by N-oxidation, in which the substrate is oxidized to nicotinamide *N*-oxide (NAM *N*-oxide) through the activity of Cytochrome P450 2E1 (CYP2E1) enzyme. In the first step of NAD^+^ biosynthesis, NAM acts as a substrate of nicotinamide phosphoribosyltransferase (NAMPT), yielding nicotinamide mononucleotide (NMN).

Nicotinamide *N*-methyltransferase (NNMT) is the enzyme that catalyzes the reaction of *N*-methylation of nicotinamide (NAM) by using S-adenosyl-L-methionine (SAM) as a methyl donor and yielding N1-methylnicotinamide (MNAM) and S-adenosyl-L-homocysteine (SAH) [30,31,32]. The methylated form of NAM may undergo urinary excretion or may be subsequently oxidized to N1-methyl-2-pyridone-5-carboxamide (2-Py) or N1-methyl-4-pyridone-3-carboxamide (4-Py), thanks to the catalytic activity exerted by aldehyde oxidase (AO) enzyme [33] (Figure 2). Analogously to MNAM, both pyridones can be excreted through urine [34].

Due to NNMT intervention to a crucial and basically irreversible catabolic reaction of NAM, enzyme activity greatly influences the regulation of intracellular levels of NAM, whose fate is represented by urinary excretion upon *N*-methylation. NAM is the amide derivative of nicotinic acid (NA) [35]. In addition to the bioactive compound of vitamin B3, NAM is also a basic precursor of nicotinamide adenine dinucleotide (NAD^+^), a pyridine nucleotide coenzyme participating in redox reactions featuring metabolism [36] and other processes, such as gene expression regulation at transcriptional level and mechanisms of DNA repair in response to structural damage [37]. Since NAM can no longer be used as an NAD^+^ precursor substrate after *N*-methylation, NNMT activity also plays a fundamental role in regulating the equilibrium between NAD^+^ biosynthesis and breakdown, thus indirectly affecting a wide range of intracellular events [35].

In addition to adipose tissue [38], liver represents the organ where the highest NNMT expression is detected. In this site, the enzyme is grouped among those participating in reactions featuring phase II metabolism and is described to be involved in the xenobiotic biotransformation and detoxification [30,31,32]. In hepatocytes, NNMT contributes to NAM homeostasis, together with other NAM-consuming enzymes, namely nicotinamide phosphoribosyltransferase (NAMPT) and Cytochrome P450 2E1 (CYP2E1); the latter is able to switch the catabolic fate of vitamin B3 towards N-oxidation rather than *N*-methylation, converting NAM into nicotinamide *N*-oxide (NAM *N*-oxide) [35,39] (Figure 2).

Studies carried out on purified human recombinant protein led to the resolution of a three-dimensional structure and to the identification of main amino acids within the active site [40]. Moreover, the enzyme kinetic mechanism was elucidated [41] and this greatly contributed to the design and testing of a variety of NNMT inhibitors [42,43,44,45,46,47,48,49,50,51,52,53,54,55,56,57,58,59,60,61,62,63].

Among human diseases in which NNMT seems to be involved, cancer displays a prominent role. In particular, enzyme upregulation has been described in renal [64], bladder [65], prostate [66], skin [67,68], thyroid [69], endometrial [70], cervical [71], ovarian [72], oral [73,74,75], nasopharyngeal [76], and lung cancers [77,78], as well as in glioblastoma multiforme [79] and in association with cancer stem cells (CSC) enrichment [80,81,82,83].

A significant bulk of scientific studies available in the literature has been focused on exploring the involvement of NNMT in association with GI cancers and speculating the enzyme contribution to GI tumorigenesis. This review summarizes data related with NNMT levels in main GI neoplasms, such as esophageal, gastric, liver, pancreatic, and colorectal cancers, as well as illustrates the main results highlighting the role played by the enzyme in different aspects of tumor cell phenotype.

## 3. NNMT and ESCC

Preliminary immunohistochemistry was carried out to explore the expression of NNMT in ESCC and adjacent normal tissue specimens. Data obtained revealed a marked enzyme overexpression in the tumor compared with the control tissue counterpart. Interestingly, a significant positive correlation was found between NNMT tumor levels and lymph node metastasis. Further analyses were performed to elucidate the role played by the enzyme in ESCC tumor cell phenotype. To this aim, siRNA-mediated NNMT silencing was achieved and the impact on proliferation, apoptosis, cell cycle, migration, and epithelial-mesenchymal transition (EMT) of EC9706 and TE1 cell lines was evaluated. Enzyme knockdown led to a reduction of cell viability and migration, as well as to cell cycle arrest and apoptosis induction. Moreover, NNMT downregulation was associated with increased expression of E-cadherin coupled with reduced levels of N-cadherin and Vimentin, demonstrating an EMT reversal [84].

In order to speculate NNMT involvement in mechanisms featuring chemosensitivity of ESCC cancer cell, three different cell lines (TE1, EC1, and Eca109) were first subjected to gas chromatography coupled with mass spectrometry (MS). Results obtained from this analysis demonstrated significant differences in nicotinamide metabolism among cell lines, with NNMT being markedly more upregulated in TE1 than in EC1 and Eca109 cells. Subsequent induction of NNMT knockdown led to a significant increase in sensitivity to treatment with 5-fluorouracil (5-FU) of TE1 cells. Moreover, in TE1 cells downregulating NNMT glucose uptake, lactate release, as well as glycolysis-related enzyme levels were decreased. On the contrary, enzyme overexpression reversed these effects in EC1 and Eca109 cells. In addition, 2-deoxyglucose-induced inhibition of glycolysis weakened the sensitivity to 5-FU promoted by NNMT. In vivo confirmation of the results obtained by in vitro experiments clearly demonstrated the ability of the enzyme to reduce 5-FU sensitivity of ESCC cells through the promotion of Warburg effect, characterized by elevated glucose consumption and fermentation of glucose to lactate [85].

## 4. NNMT and GC

By comparing different proteome profiles of GAC and matched surrounding normal tissue specimens, NNMT was identified among upregulated proteins [86,87]. These evidences gave rise to a multitude of studies focused on speculating the role played by the enzyme in GC tumorigenesis, resulting in NNMT being a promising diagnostic and prognostic biomarker, as well as molecular therapeutic target.

Subsequent gene expression analysis performed through immunohistochemistry and real-time PCR confirmed NNMT overexpression in GC compared with adjacent control tissue samples. Moreover, tumor enzyme levels were found to be positively correlated with important prognostic factors, such as primary tumor size, lymph node metastasis, distant metastasis, and tumor-node-metastasis (TNM) stage. Kaplan–Meier curves revealed that low NNMT expression levels were associated with enhanced survival rate of GC patients [88]. Subsequent elaboration of transcriptomic data highlighted the important role played by the enzyme among those metabolism-related genes, contributing to predict the prognosis of patients affected with GC [89].

Further studies were carried out to deeply investigate the prognostic potential of NNMT. In addition to being higher in GAC with respect to normal tissue and related with main prognostic parameters, enzyme expression displayed a significant positive correlation with the size of different immune cell populations surrounding the tumor, thus indicating that NNMT could promote immune infiltration of GAC. These evidences seem to suggest that patients harboring GACs with high NNMT expression may favorably respond to immunotherapeutic treatment. The sum of these results demonstrated a potential use of the enzyme as a prognostic biomarker associated with immune infiltration and a novel therapeutic target for this neoplasm [90]. The evaluation of NNMT expression was also extended to stromal cells surrounding the tumor, since they significantly contribute to cancer development and progression. Results obtained showed that stromal enzyme levels were higher in GC than in non-malignant tissue samples. In addition, NNMT intratumor expression was positively correlated with tumor stage and inversely related with patient survival, suggesting the potential use of enzyme level determination to predict the prognosis of subjects affected with GC [91].

Analyses performed in MKN28, SGC7901, MGC803, and BGC823 gastric cancer cell lines revealed that NNMT knockdown was significantly associated with the inhibition of cell proliferation, invasion, and migration in vitro, as well as tumor formation in vivo [88]. The induction of NNMT upregulation in BGC823 cells led to a significant increase of mesenchymal marked levels. The effect of modulation of enzyme levels was then evaluated in relation to transforming growth factor (TGF)-β1 expression, with this molecule playing a key role in EMT. Interestingly, results obtained clearly showed that NNMT overexpression and silencing were respectively associated with the elevated and reduced TGF-β1 levels, thus demonstrating that EMT promotion featuring GC is mediated by the upregulation of NNMT [92]. NNMT overexpression was also detected in exosomes isolated from GC patients with peritoneal metastasis (PM) compared with those without PM, as well as in exosomal vesicles obtained from GC cell line (GC-114, GC-026, MKN45, and SNU-16) with respect to GES-1 normal human gastric epithelial cells. In the context of this study, the induction of TGF-β1/Smad2 signaling mediated by NNMT expression was detected in HMrSV5 human peritoneal mesothelial cells activated by SNU-16 exosomes, indicating that GC-associated PM could be triggered by NNMT-containing exosomes, via TGF-β/smad2 signaling [93].

Two-dimensional gel electrophoresis (2-DE) followed by Western blot analyses were used to evaluate NNMT expression in tissue samples obtained from patients affected by GC and gastric ulcer. Data obtained showed the presence of a single spot in gastric ulcer tissue specimens, while four to five signals were detected in tumor tissue samples, suggesting potential GC-specific post-translational modifications of NNMT [94].

## 5. NNMT and CRC

Analyses through 2-DE coupled with MS were used to profile the gene expression of paired tumor and normal tissue specimens from CRC patients. Results obtained, confirmed by Western blot, showed that NNMT was among proteins significantly elevated in tumor compared with normal tissue. Subsequent enzyme-linked immunosorbent assay was setup to evaluate NNMT levels in serum specimens. Interestingly, enzyme levels were increased in sera from CRC patients with respect to those of controls. Further receiver operating characteristic (ROC) analysis allowed to identify the best cutoff value to discriminate CRC patients from healthy subjects and revealed an area under the curve (AUC) indicating a very good diagnostic accuracy of a serum-based NNMT test. These data seem to indicate that enzyme level determination could be used to support the early and noninvasive diagnosis of CRC [95].

A subsequent study, performed in tissue samples and in HT-29 CRAC cell line, confirmed NNMT upregulation in CRC and demonstrated that the signal transducer and activator of transcription 3 (Stat3) could serve as transcription factor promoting the expression of the enzyme [96].

Analogously to what was carried out in the context of GC, stromal NNMT expression and its prognostic value were also explored in CRC. Enzyme levels in stromal compartments of primary or metastatic CRCs were found to be more significantly increased than those detected in their respective normal-looking tissues. Moreover, stromal NNMT expression was positively related with the main unfavorable prognostic parameters, such as advanced TNM stage and the presence of lymph node/distant metastases, while a negative correlation emerged with survival of patients affected with early-stage disease. Taken together, these results make NNMT a promising biomarker for postoperative prognosis of CRC patients [97,98,99].

To elucidate the biological function exerted by the enzyme in CRC cell, as well as mechanisms featuring colorectal tumorigenesis in which NNMT seems to be involved, several studies were conducted in CRC cellular models. Variable enzyme levels were found in different human CRC cell lines, being high in HT-29 and low in SW480 cells. Therefore, NNMT overexpression and knockdown were induced in SW480 and HT-29 cells, respectively, and the impact on tumor cell phenotype was then evaluated. Results obtained showed that NNMT, as well as treatment with enzyme-methylated product MNAM, led to a significant increase of cell proliferation, migration, invasive capacity, cell cycle progression, and ATP production, together with a reduction ROS levels and inhibition of apoptosis. In addition, NNMT was able to increase in-vitro and in-vivo tumorigenicity of CRC cell lines [98,100].

By adopting an experimental approach similar to the one reported above, the effect of the enzyme on sensitivity of CRC cells to 5-fluorouracil (5-FU) treatment, and related underlying molecular mechanism, were speculated. NNMT overexpression was significantly associated with enhancement of SW480 resistance to 5-FU, while enzyme silencing led to a markedly increased sensitivity of HT-29 to chemotherapeutic treatment. In detail, enzyme expression and MNAM were able to decrease 5-FU-induced ROS formation and apoptosis induction, through the inactivation of apoptosis signal-regulating kinase 1-p38 mitogen-activated protein kinase (ASK1-p38 MAPK). Furthermore, NNMT and MNAM treatment led to a significant decrease of the inhibitory effect induced by 5-FU towards in-vivo tumor growth of CRC cells [101]. Subsequent experiments, performed by using the same cellular models, were focused on exploring the effect induced by natural compounds, known to exert antitumor activity, on modulating NNMT-associated resistance of CRC cells to 5-FU treatment. The results obtained revealed that both vanillin (Van) and curcumin (Cur), used in combination with 5-FU, were capable of inhibiting NNMT mRNA and protein levels, as well as to reverse the effects induced by enzyme expression. In particular, Van and Cur treatment reduced cell proliferation and led to a promotion of cell cycle arrest, apoptosis activation, and ROS release. Moreover, compounds administered in association with 5-FU significantly reduced CRC cell tumorigenicity, both in vitro and in vivo [102,103].

## 6. NNMT and HC

In contrast with what was reported for the vast majority of solid neoplasms, in patients affected with HCC, NNMT expression was found to be significantly decreased in the tumor compared to non-cancerous surrounding tissue, with enzyme levels being negatively correlated with tumor stage. Moreover, the survival of subjects harboring tumors displaying high enzyme expression appeared to be shorter than that of patients with reduced NNMT-immunoreactive tumors [104].

To speculate NNMT involvement in this neoplasm, a further study was carried out both in HCC cell lines and in hepatic stellate cells (HSCs), the latter residing within stroma surrounding liver parenchyma and interacting with HCC cells to promote tumor growth and metastasis. Data obtained demonstrated that the activation of HSCs led to the induction of NNMT upregulation in HCC cell lines, which in turn displayed a marked increase of migration, invasion, and metastatic potential. Subsequent analyses confirmed enzyme downregulation in HCC and demonstrated that high NNMT levels significantly correlated with main unfavorable prognostic features, as well as with poor patient survival. These results strengthened the prognostic role of the enzyme and suggested its potential use as a promising therapeutic target for HCC [105].

Western blotting and immunohistochemistry were used to evaluate the NNMT expression in tumor and normal tissue samples obtained from patients affected by CCA. Results obtained revealed a significant enzyme upregulation of CCA tissues compared with matched control specimens. The induction of NNMT overexpression in HCCC-9810 and HuCCT1 CCA cell lines was associated with an increase of in-vitro and in-vivo cell proliferation and metastatic potential. CCA progression was found to be promoted and sustained by NNMT as an effect of its catalytic activity. Indeed, as a consequence of enzyme upregulation, SAM levels undergo a significant reduction, thus inhibiting histone methylation. These statuses fosters the expression of epidermal growth factor receptor (EGFR), which in turn is able to activate the aerobic glycolysis rate [106].

## 7. NNMT and PC

The gene expression profile of pancreatic juice obtained from GC patients and healthy subjects was explored through oligonucleotide microarray analysis, followed by confirmation by real-time PCR. Results obtained revealed that NNMT mRNA levels were significantly elevated in samples of patients with GC compared to those of controls [107]. Enzyme upregulation in association with PC was then confirmed at tissue level, since NNMT expression was found to be markedly higher in PC than in pancreatic benign lesions, such as chronic pancreatitis and paracancerous tissues. Moreover, intratumor enzyme levels seem to correlate with important unfavorable clinicopathological features, with NNMT expression being increased in large-sized, late-stage, and poor differentiated tumors, thus highlighting the interesting prognostic value of the enzyme in PC [108].

NNMT knockdown in human PC cell line PANC-1 significantly reduced cell growth, migration, invasive capacity, and resistance to glucose deprivation, as well as to treatment with glycolytic inhibitor 2-deoxyglucose. Conversely, the induction of enzyme overexpression reversed the above-reported phenotypic effects, suggesting a potential involvement of NNMT in cell proliferation, metastatic potential, and resistance to metabolic stress [109].

## 8. Conclusions

**Figure 3:**
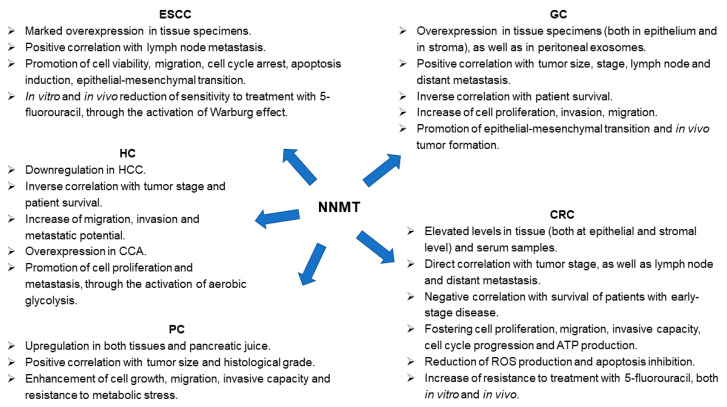
Nicotinamide *N*-methyltransferase (NNMT) role in gastrointestinal (GI) tumors. Panel illustrating differential expression (tumor versus control tissue) and enzyme involvement in cell phenotype and molecular processes featuring neoplasms affecting GI tract (ESCC, esophageal squamous cell carcinoma; GC, gastric cancer; CRC, colorectal cancer; HC, hepatic cancer; HCC, hepatocellular carcinoma; CCA, cholangiocarcinoma; PC, pancreatic cancer).

In this review, a comprehensive overview of the roles and functions exerted by NNMT in GI cancers was provided, both showing data illustrating enzyme potential as diagnostic/prognostic biomarker and reporting evidences that strongly support the possibility to make NNMT a therapeutic target (Figure 3).

For many years, NNMT was merely considered an enzyme participating in phase II metabolism, able to *N*-methylate pyridine-like compounds, mainly modulating intracellular NAM levels. However, in the last decades, a great number of studies reporting NNMT upregulation in association with cancer have been published, thus evidencing an increasing interest in the speculating function exerted by the enzyme in the context of malignant transformation [110]. Results of these works overall demonstrate that the significance of NNMT upregulation in cancer is strictly related to its ability to promote a wide range of cellular pathways and processes featuring tumor initiation and progression, such as cell proliferation, migration, invasion, and resistance to chemotherapy. In addition, a significant pro-tumorigenic effect played by NNMT was illustrated within the tumor microenvironment, such as in stromal cells [97,98,99] and in cancer stem cells [80,81,82,83].

Based on these data, NNMT started to be considered an emerging and promising novel therapeutic target for molecular anticancer treatment, leading to developing an assay of a broad number of molecules that could serve as specific NNMT inhibitors. In the context of these studies, current challenges are mainly focused on exploring/enhancing the cell permeability of these molecules, as well as on evaluating their beneficial and detrimental effects through speculations carried out in cellular and animal models [61]. The identification of such compounds, able to selectively and effectively inhibit enzyme activity, will also contribute to disclose mechanisms of action of NNMT within cancer cells and open new perspectives for therapeutic strategy development. It would be interesting to speculate whether the combination of NNMT inhibitors and traditional targeted or chemotherapeutic drugs could improve patient prognosis, fostering the future potential used of these molecules in clinical practice.

## Data Availability

Not applicable.
